# Exploration of an Integrative Prognostic Model of Radiogenomics Features With Underlying Gene Expression Patterns in Clear Cell Renal Cell Carcinoma

**DOI:** 10.3389/fonc.2021.640881

**Published:** 2021-03-08

**Authors:** Yeqian Huang, Hao Zeng, Linyan Chen, Yuling Luo, Xuelei Ma, Ye Zhao

**Affiliations:** ^1^ Department of Biotherapy, Cancer Center, West China Hospital, Sichuan University, Chengdu, China; ^2^ West China School of Medicine, West China Hospital, Sichuan University, Chengdu, China; ^3^ State Key Laboratory of Biotherapy and Cancer Center, Collaborative Innovation Center for Biotherapy, West China Hospital, Sichuan University, Chengdu, China; ^4^ School of Bioscience and Technology, Chengdu Medical College, Chengdu, China

**Keywords:** clear cell renal cell carcinoma, radiomics, genomics, machine learning, prognosis

## Abstract

**Background:**

Clear cell renal cell carcinoma (ccRCC) is one of the most common malignancies in urinary system, and radiomics has been adopted in tumor staging and prognostic evaluation in renal carcinomas. This study aimed to integrate image features of contrast-enhanced CT and underlying genomics features to predict the overall survival (OS) of ccRCC patients.

**Method:**

We extracted 107 radiomics features out of 205 patients with available CT images obtained from TCIA database and corresponding clinical and genetic information from TCGA database. LASSO-COX and SVM-RFE were employed independently as machine-learning algorithms to select prognosis-related imaging features (PRIF). Afterwards, we identified prognosis-related gene signature through WGCNA. The random forest (RF) algorithm was then applied to integrate PRIF and the genes into a combined imaging-genomics prognostic factors (IGPF) model. Furthermore, we constructed a nomogram incorporating IGPF and clinical predictors as the integrative prognostic model for ccRCC patients.

**Results:**

A total of four PRIF and four genes were identified as IGPF and were represented by corresponding risk score in RF model. The integrative IGPF model presented a better prediction performance than the PRIF model alone (average AUCs for 1-, 3-, and 5-year were 0.814 *vs.* 0.837, 0.74 *vs.* 0.806, and 0.689 *vs.* 0.751 in test set). Clinical characteristics including gender, TNM stage and IGPF were independent risk factors. The nomogram integrating clinical predictors and IGPF provided the best net benefit among the three models.

**Conclusion:**

In this study we established an integrative prognosis-related nomogram model incorporating imaging-genomic features and clinical indicators. The results indicated that IGPF may contribute to a comprehensive prognosis assessment for ccRCC patients.

## Introduction

Renal cell carcinoma (RCC) is a common heterogeneous malignancy originated from renal tubular epithelial cells, with clear cell renal cell carcinoma (ccRCC) comprising about 80% of RCC cases (1, 2). Owing to the insufficient clinical symptoms and reliable diagnostic biomarkers at the early stage, about 30% of ccRCC patients had metastasis at the time of diagnosis, and about one-fifth of patients may experience metastasis or recurrence after radical treatment (3, 4). Imageological examinations such as conventional ultrasound, contrast-enhanced ultrasound, CT, contrast-enhanced CT and MRI have been applied to assess the overall profile of the tumor as noninvasive methods. However, there are limitations in these conventional imaging tests for differential diagnosis, preoperative pathological grading and prognosis of ccRCC, which also lack quantitative criteria.

Radiomics was first proposed by Lambin et al. (5) in 2012, which exploits high-throughput feature extraction algorithms to extract quantitative image features from standard medical images. Radiomics managed to perform the conversion from images into mineable data, which could then be applied to clinical decision support systems to achieve precise prediction, diagnosis, and prognostic evaluation of cancers (6, 7). A number of studies have reported that radiomics has been successfully applied in renal tumors researches, including Fuhrman staging of ccRCC (8–10), assessment of cancer phenotype and tumor microenvironment (11), differentiation of RCC and benign renal tumors (12, 13) and efficacy and prognosis evaluation (14, 15).

However, most studies regarding radiomics were primarily focused on the selection of image features and the quantitative analysis of tumors at the macroscopic level, and there has been little research into the mechanisms of deeper molecular biology. Combined with machine learning algorithms, we can further correlate the imaging data that reflects the quantitative phenotype of the disease with the genotype feature data which reveals the molecular activity. Correlation analysis between gene mutation, expression and imaging characteristics has been proved effective in the research of liver cancer (16), lung cancer (16–18), glioblastoma (19, 20) and Alzheimer’s disease (21). Therefore, it is of vital importance to analyze the correlation and integration between imaging and genomic features of ccRCC, so as to understand the biological mechanism and furthermore obtain biomarkers for prognosis prediction, which will be more rewarding in personalized precision therapy.

Previous studies have proven that certain molecules and the activation of a series of signaling pathways are in close relation with the tumorigenesis and progression in ccRCC. For instance, the overexpression of vascular endothelial growth factor (VEGF) and platelet derived growth factor (PDGF) receptor tyrosine kinases are of great significance in promoting tumor angiogenesis and cell division. In addition, PI3K/AKT/mTOR pathway also results in affecting tumor cell growth and metabolism. Nevertheless, the associated gene expression profiles have not been thoroughly studied.

Standard treatments for ccRCC patients encompass surgery, radiotherapy and chemotherapy, and specific treatments including targeted therapy in combination with immune checkpoint inhibitors have shown efficacy in improving the overall survival (OS) of ccRCC patients (22, 23). However, the response of personalized therapy does vary and the prognosis is not as satisfactory. So far no routine genetic tests have been conducted, and these molecules concerning the mechanism of ccRCC development may provide opportunities to investigate potential biomarkers for diagnosis and prognosis. Therefore, it’s essential to establish an effective model that conduce to risk stratification, treatment strategy support and prognostic prediction for patients with ccRCC.

In this study we concentrate on analyzing the radiomics features of contrast-enhanced CT and their association with genomics profiles of ccRCC samples, which has not been extensively researched. In order to select the imaging features significantly correlated to the prognosis of ccRCC, we applied several machine learning algorithms. Through machine-learning algorithms, we further estimated the correlation between prognosis-related image features (PRIF) and expressed genes profiles. Furthermore, the integration of radiomics and gene features was conducted to enhance the accuracy of prognostic evaluation. Eventually, we conducted validation of the imaging-genomic prognostic factors (IGPF) model, and the results suggested that these features may be of help in the prediction of prognosis in ccRCC patients. The potential connection and integration of macroscopic radiomics and genetic characteristics at the microscopic level needs further exploration.

## Materials and Methods

### Data Source and Processing

**Figure 1:**
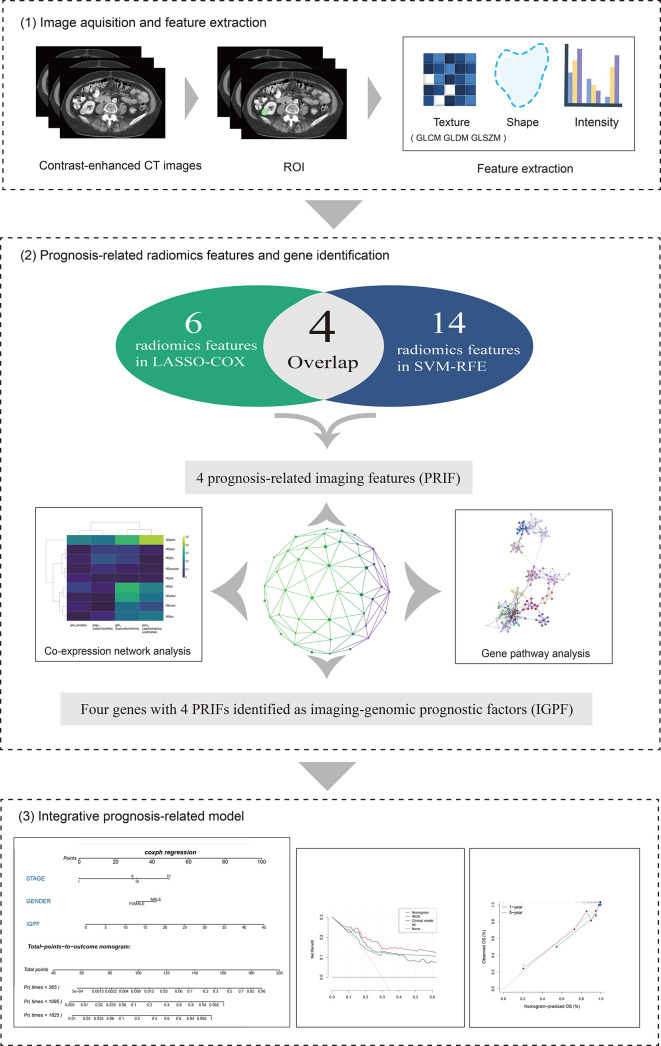
The overall framework of data analysis and model integration. 1) The segmentation of tumor region of interest (ROI) of contrast-enhanced CT images was performed by 3D slicer. Radiomics features of the ROIs were then extracted. 2) The selection of prognosis -related radiomics features was implemented by LASSO-COX Regression and SVM-RFE machine learning methods. The identification of prognostic gene modules was carried out by co-expression gene network analysis through WGCNA, and gene pathway analysis was subsequently performed. 3) The integration and assessment of prognosis-related radiomics features and gene signature was conducted by random forest (RF). Finally, the nomogram incorporating clinical predictors and imaging-genomic prognostic factors (IGPF) of ccRCC patients was constructed *via* R package rms.

The overall structure of our study was demonstrated in **Figure 1**. The detailed information of each section will be interpreted as follows. We downloaded the available enhanced CT images from the Cancer Imaging Archive (TCIA) portal (http://www.cancerimagingarchive.net/) and the information containing clinical features and mRNA sequencing data of corresponding ccRCC samples from the Cancer Genome Atlas (TCGA) database (https://portal.gdc.cancer.gov). In total 205 available samples were gathered. For data normalization, we firstly acquired the raw count data of the ccRCC patients from the TCGA-KIRC project. Then we normalized the raw count data using variance stabilizing transformation through the vst function of DESeq2 package.

### Extraction of CT Image Features

Tumor segmentation and feature extraction were performed using 3D slicer (Version 4.7) software. 3D slicer is an open source software platform which functions in medical image processing, analysis (including registration and interactive segmentation) and versatile visualization for image-guided therapy (24). We loaded deidentified transverse CT images (DICOM) of ccRCC into the software and conducted segmentation of area for each lesion with a paint function. The delineation of the region of interest (ROI) was firstly conducted by Xuelei Ma, an oncologist with experience in CT interpretation. To access the intra- and inter-rater feature stability against ROI delineation variations caused by human factors, Xuelei Ma and another experienced oncologist Ye Zhao conducted the delineation of the ROI again. Through the icc function of R package irr, we calculated the intraclass correlation coefficient and accessed the repeatability and stability of the radiomics features based on the ROI conducted by Xuelei Ma twice and that conducted by Ye Zhao (used for accessing the inter-rater stability of radiomics features).

Next we performed feature extraction calculations of ccRCC patients *via* pyradiomics package (https://pyradiomics.readthedocs.io/en/latest/), an extension *via* the 3D Slicer ExtensionManager. The pyradiomics is an open-source python package for the extraction of radiomics features from medical imaging, and most features are in compliance with feature definitions as described by the Imaging Biomarker Standardization Initiative (IBSI). Notes are added to specify the differences where the features vary in the website (25). Eventually, we obtained a total of 107 features in various classes. For instance, first order statistics describe the distribution of voxel intensities within the image region, including skewness, maximum, minimum, mean, range, and entropy etc. Shape-based category depicts shape eigenvalue of ROI and in 3-dimentional size. Gray Level Cooccurrence Matrix (GLCM) features and Gray Level Run Length Matrix (GLRLM) represent the eigenvalue of high-order texture characteristics. Other features extracted were contained in Gray Level Size Zone Matrix (GLSZM), Neighboring Gray Tone Difference Matrix (NGTDM) and Gray Level Dependence Matrix (GLDM).

### Selection of Prognosis-Related Radiomics Features

All the ccRCC samples were randomly assigned to training and test cohorts on a scale of 1:1. Based on the training set, we applied the least absolute shrinkage and selection operator COX (LASSO-COX) and support vector machines-recursive feature elimination (SVM-RFE) algorithm in R package “glmnet” and “e1071” respectively using 5-fold cross-validation approach to filtrate prognosis-related imaging features (PRIF). LASSO-COX reduces feature space dimension and filters variables by performing a penalized function that compresses insignificant coefficients to zero, and therefore contracts subsets and processes data with complex collinearity. The cv.glmnet function of glmnet package provides an argument for K-fold cross validation called “nfolds”, and this argument was set at 0.04396 for 5-fold cross validation.

SVM arranges the extracted image features in descending order according to the variable importance and inputs them to the training model in sequence in each iteration of the cross-validation calculation, thus measuring the overall accuracy of the training sets during the accumulation course. SVM-RFE is a sequence backward selection algorithm based on the maximum interval principle of SVM. We applied the 5-fold cross validation algorithm as the resampling method for SVM-RFE. The final importance of features was based on the average importance of each feature in each iteration. Afterwards, we compared the features displayed in the outcome of two methods and selected those within the intersection of two subsets as PRIF for subsequent analyses.

### Gene Co-Expression Network Analysis

To further explore the molecular biological mechanisms of the prognostic-related CT image features and obtain gene expression modules, we conducted weighted gene co-expression network analysis (WGCNA) based on training dataset. WGCNA is a systematic analytical tool which describes the correlation patterns among genes across microarray samples and clusters genes into modules, hence investigating the association between gene sets and clinical traits. The main workflow started with measuring adjacency coefficient which computes the joint strength between two nodes. Next we reduced the co-expression similarity to ensure a scale-free network. The topological overlap measure (TOM) was performed to eliminate false correlation, and then we conducted average linkage hierarchical clustering and classified functional gene modules in the co-expressed network. The module eigengenes (ME) was the first principal component of the expression matrix which represented the gene expression profile of the entire module. Afterwards we assessed the correlation between MEs and previously screened image features to identify the most relevant clinically significant module. Then to assess the preservation of the connectivity and density between each couple of modules (from the train and test networks), we carried out a permutation test through the function modulePreservation from the WGCNA package. This function provides a summary preservation Z-score for each module. Furthermore we applied Gene ontology (GO) enrichment analysis *via* Metascape (http://metascape.org) to evaluate the interlinkage between key modules.

### Construction of Integrative Imaging-Genomic Prognostic Model

We utilized random forest (RF) algorithm with 1,000 decision trees (DTs) through “randomForestSRC” (rfsrc) in R to obtain optimal prognostic factors. RF algorithm constructs and assembles multiple decision trees based on data samples to attain a more precise prediction, which can reduce the over-fitting by averaging the result. The default arguments of the rfsrc function contained a resampling method argument “bootstrap”. The default value of the “bootstrap” argument was “by.root”, which bootstraps the data by sampling with replacement at the root node before growing the tree. Based on training set we firstly constructed two prognostic models, one of which incorporated prognosis-related imaging features (PRIF) and the other integrated PRIF and the expressed genes profiles. The latter was defined as imaging-genomic prognostic factor (IGPF) model. Meanwhile we evaluated the prediction performance of the two models with test set using 5-fold cross-validation. Subsequently, we performed the discrimination of the signature by plotting the receiver operating curve (ROC) and calculating the corresponding area under curve (AUC) based on average accuracy of 5 iterations. ROC curve analysis obtained generalization abilities based on the means computed by all cross validation sets and the average 1-, 3-, and 5-year AUCs were then assessed. Furthermore, we calculated the risk scores for all ccRCC patients using RF, and patients were then separated into high-risk group and low-risk group based on the median cut-off value of risk scores. The overall survival (OS) of the two groups was acquired and displayed *via* Kaplan-Meier survival curve analysis and then compared by log rank test.

Univariate and multivariate Cox regression analyses were performed to further identify the predictive factors of survival outcome. Variables with p < 0.05 in univariate Cox regression analysis were considered statistically significant and selected for multivariate analysis. On the grounds of the results of Cox regression analysis we established a nomogram based on the training dataset, which comprised the IGPF and certain clinical factors including stage and gender through R package rms. Calibration plots were then applied based on training set to evaluate the predictive performance of the nomogram by illustrating the consistency between predicted OS and observed OS and model discrimination was estimated by the concordance index (C-index). Moreover we employed the decision curve analysis (DCA) based on training set to assess the clinical availability of the nomogram by calculating the net benefit under a range of threshold probabilities.

## Results

### Acquisition of Prognosis-Related Radiomics Features

**Figure 2:**
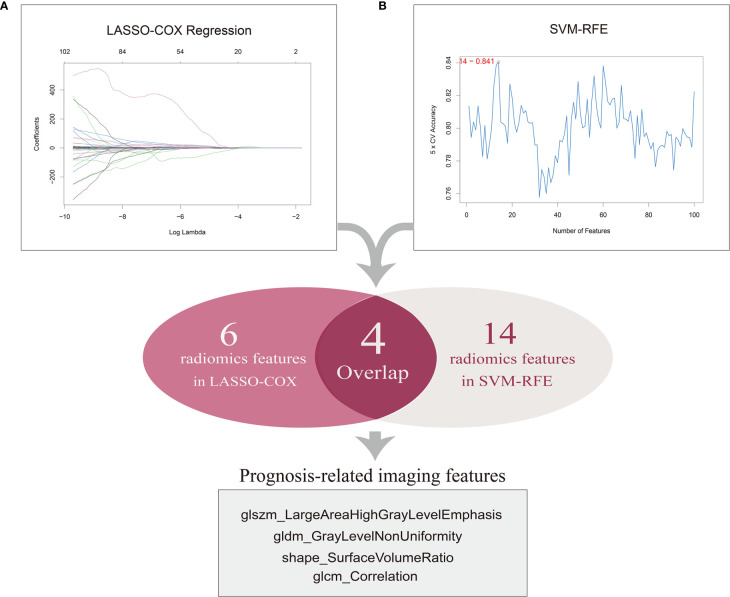
Selection of prognosis-related imaging features (PRIF). **(A)** A total of six features were identified by LASSO-COX regression analysis. The horizontal axis represents the lambda value and vertical axis represents independent variable coefficient. **(B)** A total of 14 features selected by SVM-RFE. And four imaging features within in the overlap were defined as PRIF.

We initially obtained the patient data containing clinical features and mRNA sequencing data of 537 ccRCC samples from TCGA database and the matched CT images of 237 ccRCC patients from TCIA portal, among which 205 samples with available and complete data were enrolled for subsequent analyses. The patient clinical characteristics are listed in **Table 1**. The results of the repeatability and stability assessment showed that most of the radiomics features (104 of 107) were stable against ROI delineation variations caused by human factors (icc > 0.75 and p < 0.05). The raw data of the ROI delineation by two oncologists were presented in **Supplementary Material 1**. A total of 107 features of six categories were firstly extracted from original CT images from the ROIs using pyradiomics package, and the results adhered to the IBSI recommendations (**Supplementary Material 1**, icc data). To acquire a reliable and robust model, we randomly divided the ccRCC samples into a training set (n=103) and a test set (n=102) in a 1:1 ratio and proceeded to the further selection based on the training dataset. In an attempt to diminish the possibility of module overfitting by too many radiomics features and select the ones with higher prediction accuracy for OS, two machine-learning approaches including LASSO-Cox regression and SVM-RFE were employed for mutual authentication. The tuning parameter λ was settled at an optimal value of 0.04396 with the minimum criteria in LASSO regression, and 6 prognostic features were identified with nonzero coefficients out of 107 radiomics features **(** **Figure 2A** **)**. As the extracted features ranked and excluded sequentially in SVM classifier during each iteration by contribution value, we found that the best prediction performance appeared when the first 14 radiomics features were included during the 5-fold cross validation **(** **Figure 2B** **)**.

**Table 1:** Demographic and clinical characteristics of patients.

Characteristics	Total (n=205)	Train (n=103)	Test (n=102)	P value
	NO.	NO. (%)	NO. (%)	
**Age at diagnosis(years)**				0.081
Mean ± SD	59.7 ± 12.2	58.4 ± 12.7	61.0 ± 11.7	
**Gender**				0.433
Male	134	70(68.0)	64(62.7)	
Female	71	33(32.0)	38(37.3)	
**T classification**				0.179
T1	109	56(54.4)	53(52.0)	
T2	22	15(14.6)	7(6.9)	
T3	70	31(30.1)	39(38.2)	
T4	4	1(0.9)	3(2.9)	
**N classification**				0.856
N0	83	43(41.7)	40(39.2)	
N1	5	2(1.9)	3(2.9)	
Unknown	117	58(56.4)	59(57.9)	
**M classification**				0.146
M0	176	93(90.3)	83(81.4)	
M1	28	10(9.7)	18(17.6)	
Unknown	1	0(0.0)	1(1.0)	
**TMN stage**				0.200
I	106	56(54.4)	50(49.0)	
II	18	12(11.6)	6(5.9)	
III	51	24(23.3)	27(26.5)	
IV	30	11(10.7)	19(18.6)	
**Grade**				0.227
G1	1	1(1.0)	0(0.0)	
G2	80	40(38.8)	40(39.2)	
G3	91	50(48.5)	41(40.2)	
G4	33	12(11.7)	21(20.6)	
**OS (days)**				0.090
Mean ± SD	1371.0 ± 925.1	1493.5 ± 996.7	1247.2 ± 833.5	

Therefore, the top 14 features in contribution value were filtrated as prognosis-related features for further module construction, covering six in GLCM, three in GLSZM, one in GLDM, two in shape, one in NGTDM and one in first order. Eventually four features with predictive efficiency (glszm_LargeAreaHighGrayLevelEmphasis, gldm_GrayLevelNonUniformity, shape_SurfaceVolumeRatio, glcm_Correlation) within the overlap of the results produced by the two methods were identified as prognosis-related imaging features (PRIF) **(** **Figure 2** **)**.

### Identification of Co-Expressed Gene Modules Related to Prognostic Image Features

**Figure 3:**
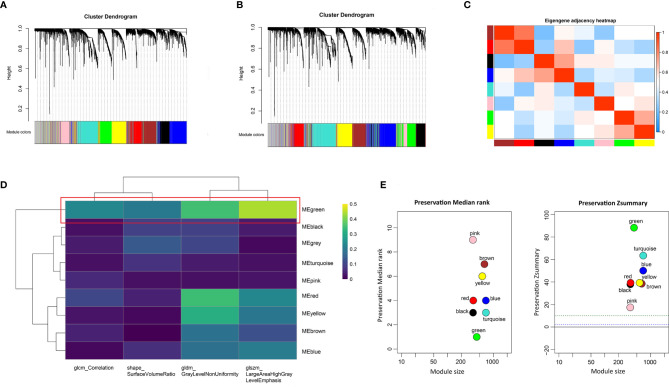
Identification of prognosis-related co-expressed gene module. **(A)** The cluster dendrogram of genes in training dataset. **(B)** The cluster dendrogram of genes in test dataset. Each branch represents one gene and each color below denotes one co-expression gene module. **(C)** Heatmap plot of relationship analysis between co-expression gene modules. **(D)** Heatmap of the correlation analysis between module eigengenes and PRIF. The green module showed the most significant correlation. **(E)** The summary preservation Z-score for each module. The higher the Z-score is, the higher the module preservation will be, whereas values below 10 indicate a moderate-to-low preservation.

To identify the gene modules highly correlated to PRIF in the ccRCC samples, we performed WGCNA to build a gene co-expression network based on training dataset. Threshold powers were set from 1 to 20 to choose an applicable soft-thresholding power, and the top 25% most variant genes (4,936 genes) ranked in descending order of SD sequence were included for subsequent analyses. A total of nine co-expressed gene modules were identified *via* the hierarchical clustering dendrogram **(** **Figures 3A, B** **)**. Relationships of the modules were illustrated in a heatmap drawn by adjacencies **(** **Figure 3C** **)**. Afterwards, we conducted correlation analysis to estimate the association between nine MEs and image traits **(** **Figure 3D** **)**. The correlation coefficients and FDR values between each of the nine gene modules and PRIF were displayed in **Supplementary Material 2**. Of all the nine gene co-expression modules, the green module (625 genes) displayed the most significant correlation with the prognosis-related image features of ccRCC, including glszm_LargeAreaHighGrayLevelEmphasis, gldm_GrayLevelNonUniformity, shape_SurfaceVolumeRatio and glcm_Correlation. The module preservation analysis presented by the summary preservation Z-score showed that all the modules were rather stable and the green module was the most robust between training and test sets **(** **Figure 3E** **).** Thus we identified the green module as the key module of significant prognostic importance for continuous research.

**Figure 4:**
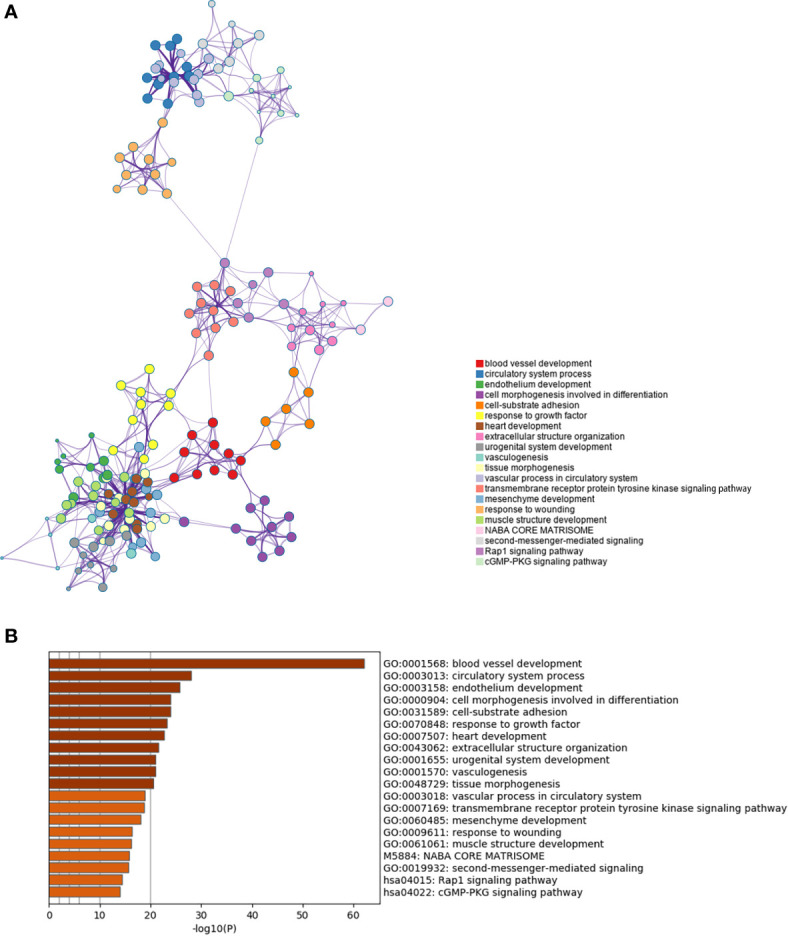
Enrichment analysis of the prognosis-related gene co-expression green module. **(A)** Metascape enrichment network visualization cluster of genes in green module. Each circle node denotes one term and the color of node indicates its cluster identity, representing the intra-cluster and inter-cluster similarities of enriched terms. Cluster annotations and the most significantly enriched terms are shown in color code. **(B)** GO enrichment analysis of the co-expressed genes in green module.

Furthermore we carried out enrichment analysis to describe the biological interpretations of the genes in green module **(** **Supplementary Material 3** **)**. As illustrated in **Figure 4**, the genes were significantly related to certain biological processes such as blood vessel development, circulatory system process, cell morphogenesis involved in differentiation, cell-substrate adhesion, and extracellular structure organization. The results suggested that these genes may be involved in tumor angiogenesis and cell adhesion, which have been proved to be associated with tumorigenesis and progression.

### Construction and Validation of Integrated Imaging-Genomic Prognostic Model

In order to establish an integrative model of PRIF and prognostic co-expressed gene profile, we applied RF algorithm based on training dataset, and furthermore performed model verification with the test dataset. Initially we presented PRIF as an independent variable to analyze its impact on prognosis and found a significant correlation. Then to explore the combined effect of genomics and imaging features, we assessed gene expression profiles in the prognostic-related green module and selected the top four genes with the highest module membership (MM) value (RPS6KA2, CYYR1, KDR, GIMAP6) **(** **Supplementary Material 4**, **Figure S1** **)**.

**Figure 5:**
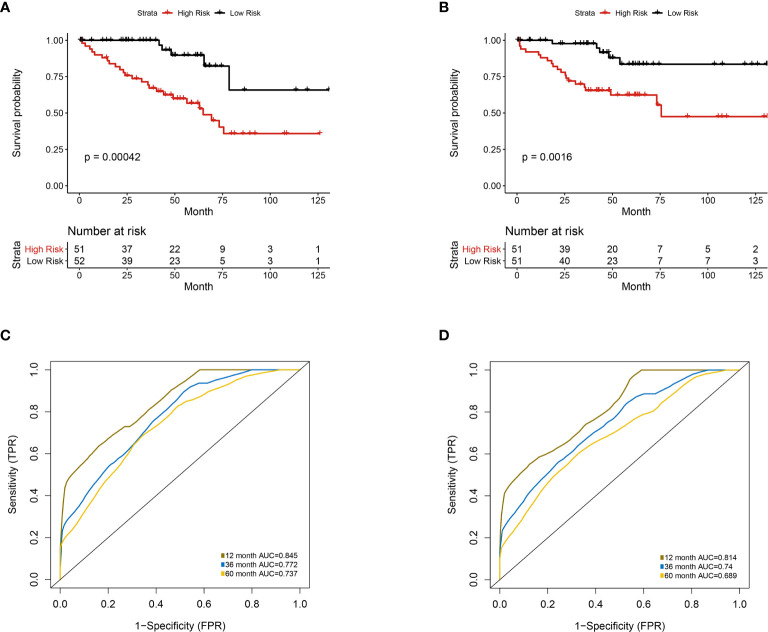
Univariate analysis of prognosis-related radiomics features model. Patients were divided into high-risk group and low-risk group according to the median value of IGPF risk score. **(A, B)** Kaplan-Meier curves demonstrating overall survival (OS) of patients in high-risk group and low-risk group in **(A)** training set and **(B)** test set. **(C, D)** The 1-, 3-, and 5-year area under curve (AUC) of receiver operating curve (ROC) in **(C)** training set and **(D)** validation test set.

**Figure 6:**
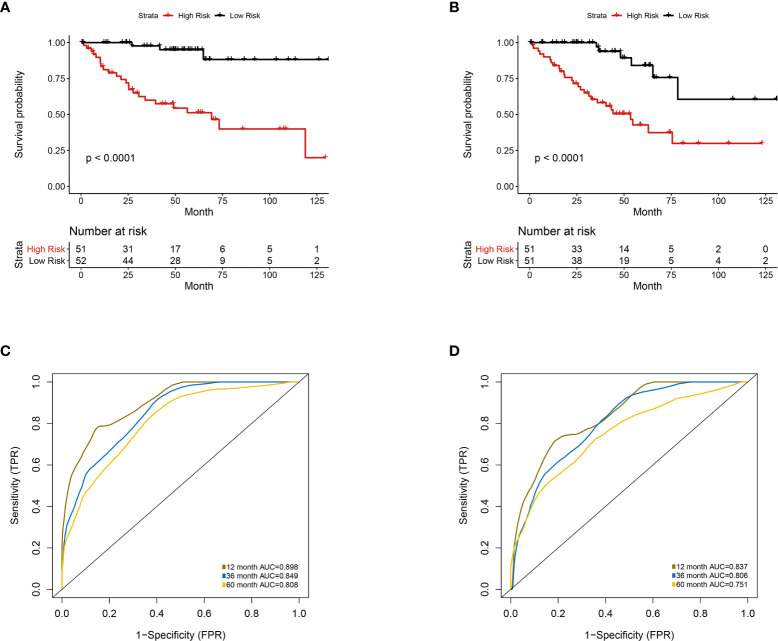
Multivariate analysis of the integrative prognostic model incorporating radiomics and genomics features. Patients were divided into high-risk group and low-risk group according to the median value of IGPF risk score. **(A, B)** Kaplan-Meier curves demonstrating OS of patients in high-risk group and low-risk group in **(A)** training set and **(B)** test set. **(C, D).** The 1-, 3-, and 5-year area under curve (AUC) of receiver operating curve (ROC) in **(C)** training set and **(D)** validation test set.

Furthermore, we integrated the four genes with PRIF which were identified as imaging-genomic prognostic factors (IGPF) and calculated the risk score of each ccRCC patient. The patients were divided into high-risk and low-risk groups in light of the median value of risk scores and then estimated with time-dependent ROC. To evaluate the statistical differences between different models, we applied the compare function of timeROC package in both training and test sets. The result showed that there were statistically significant differences between RPIF and IGPF models in 1-, 3-, and 5-year OS (P<0.05) **(** **Table 2** **)**. The outcome illustrated a more satisfactory predictive performance of IGPF model compared to the RPIF model alone **(** **Table 3** **)**. In the training set, the average AUCs for 1-, 3-, and 5-year OS were 0.845, 0.772, and 0.737 in PRIF model compared to 0.898, 0.849 and 0.808 in IGPF model respectively **(** **Figures 5C**, **6C** **)**. In the test set, the average AUCs for 1-, 3-, and 5-year OS were 0.814, 0.74 and 0.689 of PRIF model compared to 0.837, 0.806 and 0.751 of the combined IGPF module **(** **Figures 5D**, **6D** **)**.

**Table 2:** Comparison of PRIF and IGPF models in training set and test set.

Dataset	Time (d)	P value
**Train** (n=103)	t=365	0.294493777
	t=1095	0.012522423
	t=1825	0.006498863
**Test** (n=102)	t=365	0.048720526
	t=1095	0.02381105
	t=1825	0.007957811

PRIF, prognosis-related imaging features; IGPF, imaging-genomic prognostic factors.

**Table 3:** Survival models based on PRIF and IGPF in training set and test set.

Model		HR	z	P value	lower	upper	c-index	se(C-index)
**Train** (n=103)	IGPF	9.555645221	3.663583845	0.000248711	2.856506317	31.96574608	0.7435393	0.0303249
	PRIF	5.890757826	3.171763552	0.001215163	2.833927216	14.04278158	0.68764045	0.04130209
**Test** (n=102)	IGPF	7.624785255	4.189170631	0.000027998	2.947573776	19.72379815	0.74161074	0.02908009
	PRIF	4.461795265	3.522355167	0.000427731	1.941329799	10.25462907	0.68504314	0.03922711

PRIF, prognosis-related imaging features; IGPF, imaging-genomic prognostic factors.

### Establishment and Evaluation of Nomogram Model

**Figure 7:**
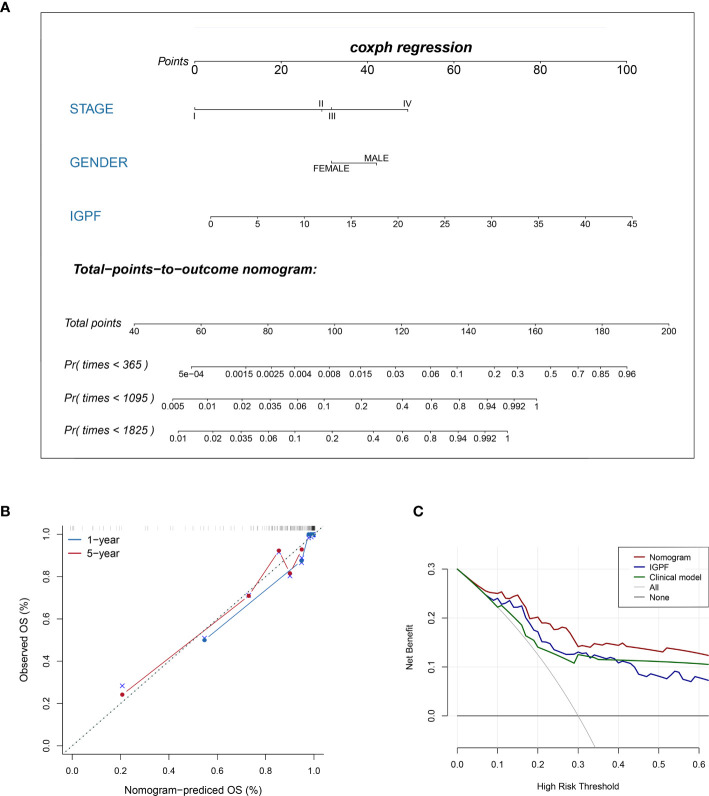
Construction and validation of prognostic nomogram model. **(A)** The nomogram prediction of the 1-, 3-, and 5-year OS of ccRCC patients. **(B)** Calibration plots of the nomogram for 1- and 5-year OS prediction. The horizontal axis represents nomogram-predicted survival probability and the vertical axis represents actual survival. **(C)** Decision curve analyses of IGPF, clinical and nomogram model. The gray oblique line represents the net benefit of all intervening patients, and the horizontal gray line indicates the net benefit of no intervening patients. The nomogram model showed the best net benefits in the vast majority of the threshold probability range.

According to Kaplan-Meier survival curves, a distinctly significant difference of p < 0.0001 can be seen between the two groups in both test and train cohorts, and patients in the low-risk group showed a more promising OS than the high-risk group **(** **Figures 5A, B**, **6A, B** **)**. In consideration of the relationship of IGPF and certain clinical predictors, we performed univariate and multivariate Cox analysis. The results indicated that clinical characteristics including gender, TNM stage and IGPF were independent risk factors for OS of ccRCC patients. In order to acquire a quantitative prediction method for disease progression and survival probability of ccRCC, we established a nomogram on the basis of the independent predictors of OS (gender, TNM stage, and IGPF) identified earlier **(** **Figure 7A** **)**. Calibration plots were then applied to assess the consistency between the nomogram-predicted values and actual values, and the calibration curves in **Figure 7B** denoted good performance of 1- and 5-year nomogram model which showed a closer tendency to the 45-degree standard line. Meanwhile, the decision curves analysis evaluated the clinical utility of IGPF model containing radiomics and gene features, clinical model that involved TNM stage and gender and nomogram which integrated the former two models **(** **Figure 7C** **)**. As depicted in the results, nomogram provided the best net benefit among most of the threshold probabilities range.

## Discussion

In this study, we extracted radiomics features from contrast-enhanced CT images of ccRCC, and subsequently selected prognosis-related image features (PRIF) with significant prognostic value *via* several machine-learning algorithms. Furthermore we identified gene modules that are most relevant to PRIF through co-expression network. Based on the PRIF (screened by LASSO and SVM-RFE) and genes (screened by WGCNA and MM value), we constructed a robust imaging-genomic prognostic factors (IGPF) model incorporating prediction features in the two categories through random survival forest algorithm. The random survival forest algorithm acts as a bootstrap algorithm and can predict the overall survival. The OS prediction analysis demonstrated a notable performance of the integrative prognostic model, and thus the IGPF based risk score was considered as an independent prognostic factor. Afterwards, through nomogram we integrated the IGPF model and clinical predictor model, and then made comparisons of the three prognostic models. Ultimately, the prediction capability of the nomogram model outweighed the other two.

On the basis of the initially obtained 107 imaging features, we employed two machine-learning methods LASSO-Cox and SVM-RFE in combination aiming to achieve a group of prognostic radiomics features with more robust and accurate prediction abilities. Four conspicuous prognosis-related image features in our study were included in Gray Level Size Zone Matrix (GLSZM), Gray Level Dependence Matrix (GLDM), shape and Gray Level Cooccurrence Matrix (GLCM) respectively. As illustrated in the results, features based on intensity discretization were not screened out in the end. The results suggested that under these two unsupervised feature selection algorithms, the gray level-based features and shape-based features had a better prognostic performance than intensity discretization-based features in this cohort. However, considering the differences and limitations among multiple algorithms and cohorts, we cannot completely deny the importance of intensity discretization-based features.

A gray level zone is described as the number of connected voxels which show the same intensity. The texture feature Large Area High Gray Level Emphasis from GLSZM quantifies the proportion in the image of the joint distribution of smaller size zones with higher gray-level values, which has been formerly adopted in the assessment of the robustness or patient response in different imageological examinations (26, 27). The GLDM-based textural feature Gray Level Non Uniformity (GLN) calculates the similarity of gray-level intensity values, where a lower GLN refers to a higher intensity value in the image (28). Surface Area to Volume Ratio is a shape feature that is not dimensionless and is partly dependent on the volume of the ROI. It has been utilized in differentiating the benign and malignant tumors based on shape and margin of the lesions (29, 30). GLCM conduces to reflecting the comprehensive information about pixel distribution containing direction, distance, gray value, and the pattern of gray level arrangement (28), and Correlation represents the linear dependency of gray level values to their respective voxels in the GLCM textural features. It has been applied previously in the evaluation of breast cancer, osteosarcoma, lung cancer and gliomas in imaging modalities such as CT, MRI, and PECT (31–35).

In our study, the predictive efficacy of the elected prognostic related radiomics features based on training set were found to be in accordance with some of the reference research above (30, 33, 34, 36). However, a lot of former studies have concentrated on the performance of textural features of radiographic images, which may lack a comprehensive explanation of the biological mechanism and potential biomolecular features of the disease. While in our study, we conducted the identification of the prognostic gene co-expression module and then evaluated the association between the imaging phenotype and genomic characteristics. The results demonstrated that the green module was most related to all the PRIF, and gldm_gray level non uniformity feature could be mostly affected by gene expression pattern. In addition, the red and yellow modules also had a relatively high correlation with the gldm_gray level non uniformity feature. This may be related to the objective attributes of this feature, and further studies are still needed to explain the potential relevance and biological mechanism between gene modules and radiomics features. Moreover, we implemented enrichment analysis in order to elaborate the latent molecular pathways relevant to the prognostic significant green gene module.

The results indicated that the most prominent enrichment leans towards pathways involved in tumor angiogenesis, cell adhesion and extracellular structure organization. Formation of new vascular networks is a pivotal step in tumor progression and also expedites the metastasis of cancer cells (37). At present, tumor microvessel density (MVD) and VEGF are important immunohistochemical indicators for tumor angiogenesis, and studies have reported that three-phase dynamic enhanced CT and MRI can be utilized as auxiliary evaluation methods for tumor angiogenesis, malignancy and prognosis in ccRCC (38–40). Cell-substrate adhesion has been widely demonstrated as an indispensable process of metastasis *in vivo* (41). The modification of cell adhesion status has significant impact on biophysical patterns of tumor microenvironment (TME) and structure of extracellular matrix (ECM), which has been reported to be related to the prognosis of colorectal cancer, lung cancer and gastric cancer (42–45). In accordance with previous researches, the results may provide a chance to understand the upstream biological mechanisms of tumor development in ccRCC (46–48). RPS6KA2, CYYR1, KDR, and GIMAP6 were discovered to be most correlated with the prognostic-related module eigengene, which was also found relevant to blood vessel development and cell proliferation in existing researches. For instance, KDR has been reported to acts as an important mediator of VEGF-induced endothelial proliferation, tubular morphogenesis and sprouting and associate with signaling by GPCR pathway (49, 50). RPS6KA2 has been found to act downstream of EGFR, RAS, and ERK signaling, which mediates mitogenic and stress-induced activation of transcription factors and thus regulate the proliferation and differentiation of cells (51, 52).

Subsequently, we integrated the prognosis-related image features and gene profiles into an IGPF model and obtained corresponding risk scores. The clinical model took in gender and TNM stage as the common tumor assessment indicators for prognosis, but the predictive accuracy is still limited. The nomogram which integrated IGPF and clinical predictors was validated to outperform all the models with the best prediction performance.

There were several limitations to this study. First of all, the sample size was comparatively small because patients with available identified transverse CT images and gene expression profiles were limited. Secondly, the data of patients we enrolled may be incomplete, which might create discrepancies and lead to potential bias. To better promote the conclusions and understand the underlying biology molecular mechanism, a larger scale of multi-center data verification is necessarily needed. Thirdly, since we used random survival forest algorithm to build survival prognosis model in this study, the bootstrap step was a built-in process and the bootstrap corrected results could not be reported. Fourthly, more clinical trials and experimental researches are needed to assess the prove the adaptability of the imaging-genomic prognostic model, and the molecular mechanisms remain to be further explored.

In conclusion, in this study we constructed an integrative prognosis-related model incorporating radiomics features, genomic profile and clinical indicators. The results illustrated that IGPF may improve the prognostic modalities on the basis of conventional clinical indexes, and the nomogram prediction model can serve as an advantageous measurement tool which may be conducive to personalized treatment and prognosis for ccRCC patients.

## Data Availability Statement

Publicly available datasets were analyzed in this study. This data can be found here: http://www.cancerimagingarchive.net/; https://portal.gdc.cancer.gov.

## Author Contributions

YH: data curation, writing – original draft and submission. HZ: conceptualization, methodology, and software. LC: validation, writing – reviewing and editing. YL: writing – reviewing and editing. XM: conceptualization and supervision. YZ: conceptualization and supervision. All authors contributed to the article and approved the submitted version.

## Funding

The study was funded by the National Natural Science Foundation of China, grant no. 31701212.

## Conflict of Interest

The authors declare that the research was conducted in the absence of any commercial or financial relationships that could be construed as a potential conflict of interest.
