# Supplementary figures and images for: Exploration of an Integrative Prognostic Model of Radiogenomics Features With Underlying Gene Expression Patterns in Clear Cell Renal Cell Carcinoma
Source: Front Oncol. 2021 Mar 8;11:640881. doi: 10.3389/fonc.2021.640881 (PMC7982462; doi:10.3389/fonc.2021.640881)

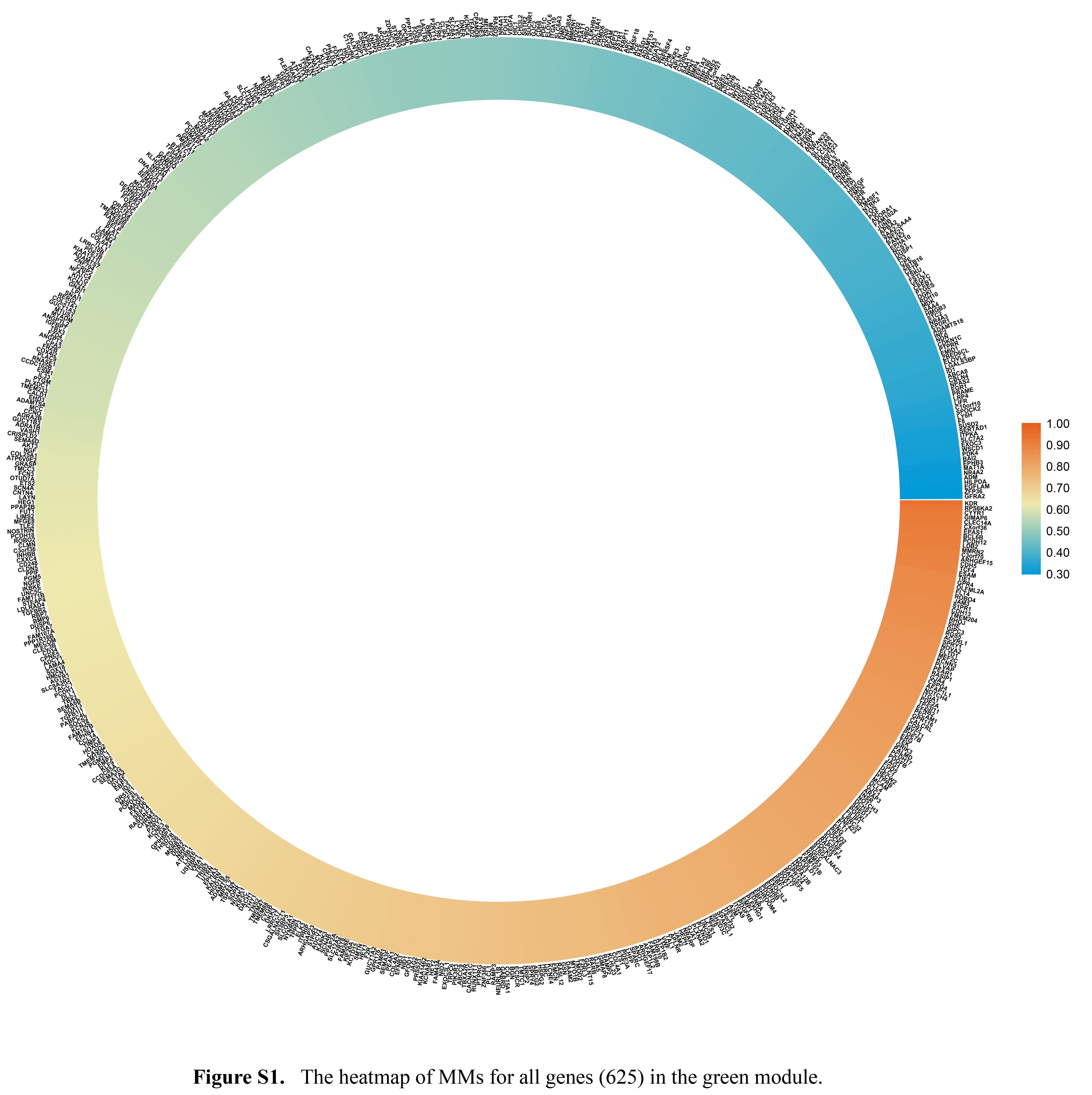

Supplement: Supplementary file 5 [file Image_1.tif]
